# Unveiling the molecular mechanisms of burn injury through integrated single-cell and bulk transcriptomic analysis

**DOI:** 10.1371/journal.pone.0341725

**Published:** 2026-01-27

**Authors:** Xiaoyu Zhu, Neng Huang, Yanyan Pan, Xin Le, Shengyong Cui, Jiliang Li, Youfen Fan

**Affiliations:** 1 School of Medicine, Ningbo University, Ningbo, Zhejiang, China; 2 Ningbo No. 2 Hospital, Ningbo, Zhejiang Province, China; Xiangya Hospital Central South University, CHINA

## Abstract

**Purpose:**

Burn injuries are prevalent and have a significant effect on patients’ quality of life and healthcare costs. Current treatment modalities, such as wound care and surgical interventions, often face challenges due to complications like infection and inadequate healing.

**Methods:**

This study adopted single-cell RNA sequencing (scRNA-seq) to investigate the cellular landscape of the burn microenvironment. After rigorous quality control filtering, 9,248 cells were retained for analysis. Using UMAP dimensionality reduction, these cells were annotated into 14 subpopulations, including Neutrophils, Macrophages, and T cells. Differential gene analysis and machine learning techniques, such as LASSO regression and random forest selection, were employed to identify marker genes.

**Results:**

Macrophages exhibited significant interactions with other cell types, indicating their pivotal role in immune signaling within the burn microenvironment. A total of 155 genes were identified as markers for Macrophages, with AP2A2, CCL7, and TF emerging as key features. Immune infiltration analysis also revealed notable differences in the proportions of immune cells, particularly Mast cells and Neutrophils, highlighting on their involvement in disease progression.

**Conclusion:**

This study provides novel insights into the immunological microenvironment of burn injuries. Despite limitations including a modest sample size and lack of experimental validation, our findings establish a foundation for future investigations into targeted immunotherapeutic strategies, potentially improving clinical outcomes and advancing personalized treatment approaches for burn patients.

## Introduction

Burns represent not only a significant public health concern but also inflict profound physical and psychological distress upon affected individuals, thereby imposing considerable economic burdens on both the healthcare system and society at large [1]. Their elevated incidence rates compel healthcare professionals to allocate extensive resources, including long-term rehabilitation and specialized treatment. These injuries and their consequences markedly compromise the quality of life for survivors [2]. The intricate clinical nature of burns and their repercussions on various physiological systems cannot be overlooked. Beyond the immediate destruction of the skin, burns can trigger numerous systemic responses, potentially leading to multi-organ dysfunction. Contemporary therapeutic approaches, including wound management, analgesia, and surgical intervention, are frequently impeded by protracted healing periods and complications such as infections, highlighting the urgent need for innovative treatment modalities [3].

The clinical management of burn injury centers on mitigating systemic inflammatory response syndrome, sepsis, multiple organ failure, and other potentially fatal conditions [4]. Burns represent a highly intricate pathological phenomenon, frequently characterized by significant fluid depletion, pronounced inflammation, elevated metabolic activity, and acute immune responses [5,6]. Research has indicated that neutrophils are pivotal in the initial phases of burn injury due to their involvement in phagocytosis, degranulation, and the liberation of reactive oxygen species (ROS) [7–10]. While advancements have been achieved in deciphering the immune mechanisms involved in wound healing, the intricate interplay between immune cell dynamics and burn progression remains inadequately elucidated. However, conventional research methodologies exhibit limitations in uncovering the underlying mechanisms associated with burn injury. Prior investigations predominantly adopted a macro-level perspective, failing to explore the specific cellular and molecular mechanisms at play. The advent of cutting-edge technologies, such as single-cell sequencing and transcriptomics, has ushered in novel insights [11–13]. These technologies enable the analysis of cellular heterogeneity and dynamic alterations at the single-cell level, thereby providing crucial understanding of the biological responses following burn injury. This study aspires to bridge the existing knowledge gap by examining the regulatory interactions between key genes and immune cells in the context of burn injury, which will aid in the formulation of more effective therapeutic strategies.

This investigation harnesses state-of-the-art single-cell RNA sequencing technology to meticulously dissect the cellular environment and immune interaction network associated with burns [14]. Consequently, the paramount benefit of this approach lies in its ability to unveil cellular heterogeneity and the dynamic interplay among diverse immune cell types with unparalleled resolution. The research delves into the intricate associations between pivotal genes and immune cell interactions in the context of burn injuries; this area has historically posed significant challenges for patient recovery and healthcare systems. Notably, we have, for the first time, identified three essential genes—AP2A2, CCL7, and TF—that exhibit a strong correlation with immune dynamics, particularly regarding the function of macrophages, which are recognized for their critical roles in wound healing and inflammatory responses. The associations uncovered in this study underscore the vital importance of immune regulation networks in burn therapy and furnish robust evidence for further investigation into the clinical applicability of these immune signaling pathways.

## Methods

### Data download

The NCBI GEO (https://www.ncbi.nlm.nih.gov/geo/), short for GENE EXPRESSION OMNIBUS, is a gene expression database created and maintained by the National Center for Biotechnology Information (NCBI). Single-cell data files for GSE163446 were downloaded from the NCBI GEO public database. Data from three samples with complete single-cell expression profiles were downloaded for single-cell analysis. The Series Matrix File for GSE139028, with annotation file GPL15433, was also downloaded from the NCBI GEO public database. Expression profile data from nine patients were included: three in the control group and six in the disease group. Our analysis used publicly available summary statistics. No new data were collected, and no new ethical approval was required.

### Quality control

First, expression profiles were imported using the Seurat package. Cells were filtered based on the total number of UMIs per cell, the number of expressed genes, and the percentage of mitochondrial and ribosomal reads per cell. Outliers were defined as the median absolute deviation (MAD) of three from the median. Cells with excessively high total UMIs and expressed gene counts are generally considered diploid cells. Cells with excessively high mitochondrial read percentages and ribosomal read percentages are considered to be of poor quality and on the verge of apoptosis or fragmentation. As we utilized publicly available databases, no new ethical approval was required for this study.

### Data normalization and cell annotation

Data were normalized using the NormalizeData function, cell cycle scores were calculated using CellCycleScoring, highly variable genes were identified using FindVariableFeatures, and data were normalized using ScaleData to eliminate the influence of mitochondrial, ribosomal, and cell cycle genes on subsequent analysis. RunPCA performed linear dimensionality reduction on the expression matrix and selected principal components for subsequent analysis. Harmony was used to remove batch effects. It iteratively clusters similar cells from different batches in PCA space while maintaining the diversity of batches within each cluster. RunUMAP (Uniform Manifold Approximation and Projection) was used for nonlinear dimensionality reduction. FindNeighbors was used to identify cell neighbors, and FindClusters was used to group cells into clusters. Cell annotation was performed by querying the CellMarker database and literature, supplemented by automated annotation using SingleR software, to identify cell types and corresponding marker genes present in the corresponding tissue.

### Ligand-receptor interaction analysis (CellChat)

CellChat is a tool that quantitatively infers and analyzes intercellular communication networks from single-cell data. It uses network analysis and pattern recognition methods to predict the primary signaling inputs and outputs of cells and how these cells and signals coordinate their functions. In this analysis, we used standardized single-cell expression profiles as input data and cell subtypes derived from single-cell analysis as cell information. We analyzed cell-related interactions and quantified the closeness of these interactions using interaction strengths (weights) and counts, thereby examining the activity and impact of each cell type in disease.

### Lasso regression and random forest feature selection

We used lasso logistic regression and random forest algorithms for feature selection of disease diagnostic markers. The lasso algorithm was implemented using the “glmnet” package. Random forest is an ensemble learning algorithm based on decision trees. Using sampling with replacement, multiple samples are selected from a sample set as a training set, and a decision tree is generated using the sampled set. At each generated node, features are randomly selected without duplication. These features are used to partition the sample set, identify the optimal partitioning features, and determine the prediction results. This study evaluated feature importance using the random forest algorithm and %IncMSE, and selected the top 10 features for subsequent analysis.

### Immune infiltration

The CIBERSORT method is a widely used method for evaluating immune cell types within the microenvironment. Based on the principles of support vector regression, this method performs deconvolution analysis on immune cell subtype expression matrices. It contains 547 biomarkers that distinguish 22 human immune cell phenotypes, including T cell, B cell, plasma cell, and myeloid cell subsets. This study used the CIBERSORT algorithm to analyze sample data, inferring the relative proportions of the 22 immune infiltrating cell types and performing correlation analysis between gene expression and immune cell content.

### GSEA analysis

Based on the expression levels of key genes, samples were divided into high- and low-expression groups. GSEA was then used to further analyze differences in signaling pathways between the two groups. Background gene sets, version 7.0, were downloaded from the MsgDB database as annotation gene sets for subtype pathways. Differential expression analysis of pathways between different groups was performed, and significantly enriched gene sets (adjusted p-value less than 0.05) were ranked based on consistency scores. GSEA analysis is often used to closely integrate disease classification with biological significance.

### GSVA analysis

Gene set variation analysis (GSVA) is a nonparametric, unsupervised method for assessing transcriptome gene set enrichment. GSVA uses a comprehensive score for gene sets of interest to translate gene-level changes into pathway-level changes, thereby assessing the biological function of the sample. In this study, gene sets were downloaded from the Molecular Signatures database and the GSVA algorithm was used to comprehensively score each gene set to assess potential biological function changes across samples.

### Key gene-associated noncoding RNA network

miRNAs are small noncoding RNAs that have been shown to regulate gene expression by promoting the degradation or inhibiting the translation of mRNAs. Therefore, we further analyzed whether a subset of miRNAs in key genes regulates the transcription or degradation of risk genes. We obtained miRNAs associated with key genes from the mircode database and visualized the miRNA network of these genes using cytoscape software.

### Transcription factor regulatory network

This study used the R package “RcisTarget” to predict transcription factors. All calculations performed by RcisTarget are motif-based. The normalized enrichment score (NES) of a motif is determined by the total number of motifs in the database. In addition to motifs annotated by the source data, we also inferred further annotation files based on motif similarity and gene sequence. The first step in estimating the overrepresentation of each motif in a gene set is to calculate the area under the curve (AUC) for each motif-motif set pair. This is performed based on the recovery curve of the gene set-ranked motifs. The NES for each motif is calculated based on the distribution of AUCs across all motifs in the gene set.

### Drug prediction

The Comparative Toxicogenomics Database (CTD) integrates a large amount of interaction data between chemicals, genes, functional phenotypes, and diseases, greatly facilitating the study of disease-related environmental exposures and potential drug mechanisms of action. We searched the CTD to predict potential drugs or molecular compounds that interact with key genes. The database contains over 46.64 million interaction data points, encompassing over 2.3 million chemicals, 46,689 genes, 4,340 phenotypes, and 7,212 diseases.

### Statistical analysis

This analysis was conducted using R (version 4.3.0). All statistical tests were two-sided, and p < 0.05 was considered statistically significant.

## Results

### Single-cell data quality control

Considering data quality across multiple samples, outliers and cells with fewer than 200 genes were filtered out, resulting in a total of 9,248 cells retained. Violin plots and scatter plots after filtering are shown (S1 Fig). We then analyzed 2,000 highly variable genes, and the 10 genes with the highest standard deviations are displayed (S2A Fig). The data were then subjected to standardization, homogenization, PCA, and Harmony analysis (S2B, C Fig).

### Cell annotation

**Fig 1:**
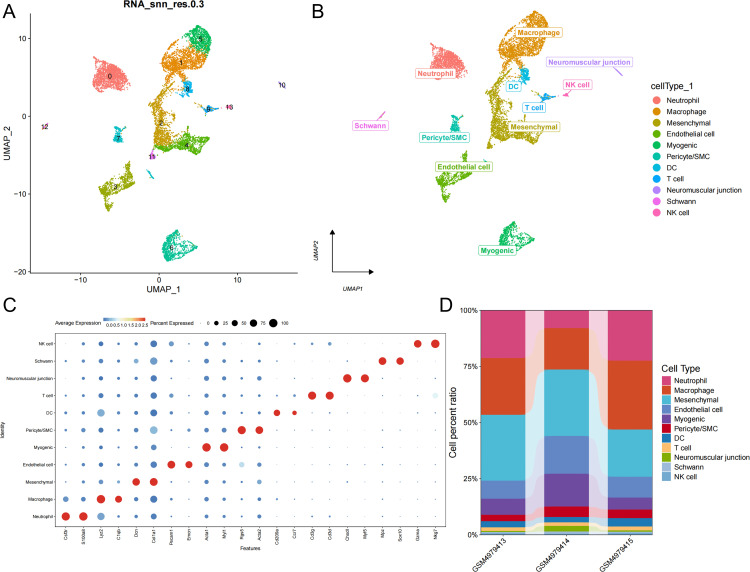
Cell Type Annotation and Gene Expression Analysis of Single-Cell RNA Sequencing Data. A. UMAP visualization of single-cell data. B. Projected Expression of Immune Cell Subtypes on UMAP. C. Bubble chart of gene expression across 11 cell subtypes. D. The proportion of cell subpopulations in different datasets.

After dimensionality reduction using the Unified Manifold Approximation and Projection (UMAP) method, 14 subpopulations were identified. This study annotated these cells using known cell markers, resulting in 11 cell types: neutrophil, macrophage, mesenchymal, endothelial cell, myogenic, pericyte/SMC, DC, T cell, neuromuscular junction, Schwann cell, and NK cell (Fig 1A, B). The expression of key marker genes for 11 cell types was visualized using bubble plots (Fig 1C), and histograms of the cell proportions corresponding to these 11 cell types across different samples were also plotted (Fig 1C, D).

### Cell communication and subpopulation differential enrichment analysis

**Fig 2:**
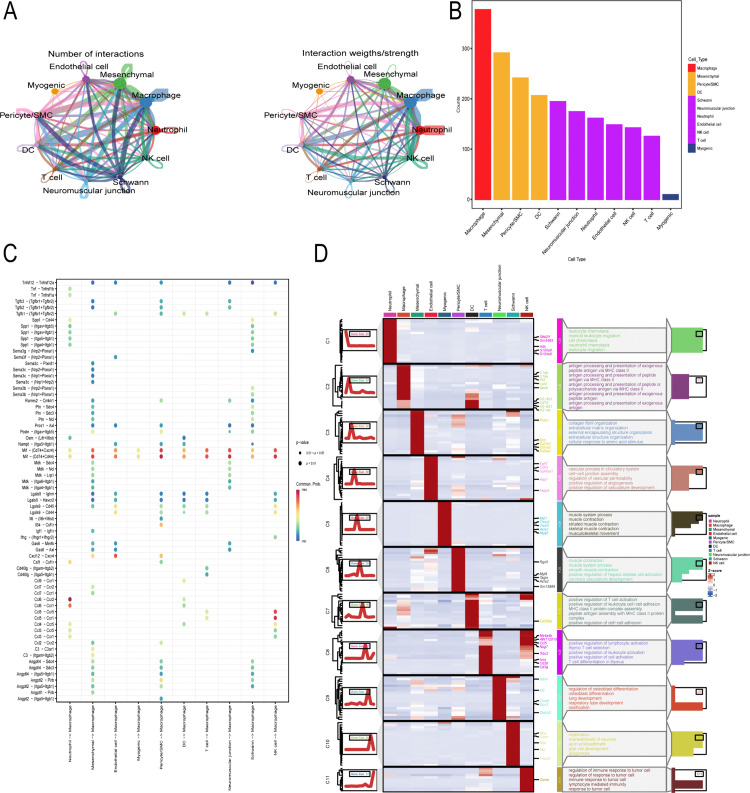
Intercellular Crosstalk and Gene Expression Pattern Analysis. A. Intercellular communication between different cell types. B. Connectivity of Different Cell Types in Networks. C. Bubble chart illustrating the significance of communication between different cell types. D. Enrichment Heatmaps and Annotations for Average Expression Across Cell Subpopulations.

We used the software package cellchat to analyze ligand-receptor relationships among genes in the single-cell expression profiles. We discovered complex interactions between these cell subtypes (Fig 2A). Finally, we statistically identified macrophages as having more potential interactions with other cells and demonstrated that these cells, when acting as signaling targets, are ligand receptors for other cells. Therefore, we identified macrophages as key cells for our study (Fig 2B, C). Next, we performed subpopulation differential expression and enrichment analysis, using ClusterGVis to display enrichment heatmaps and annotations of average expression in cell subpopulations (Fig 2D). The results showed that differentially expressed genes in macrophages were enriched in pathways such as antigen processing and presentation of exogenous peptide antigens via MHC class II, antigen processing and presentation of peptide antigens via MHC class II, and antigen processing and presentation of peptide or polysaccharide antigens via MHC class II.

### Lasso regression and random forest feature selection

**Fig 3:**
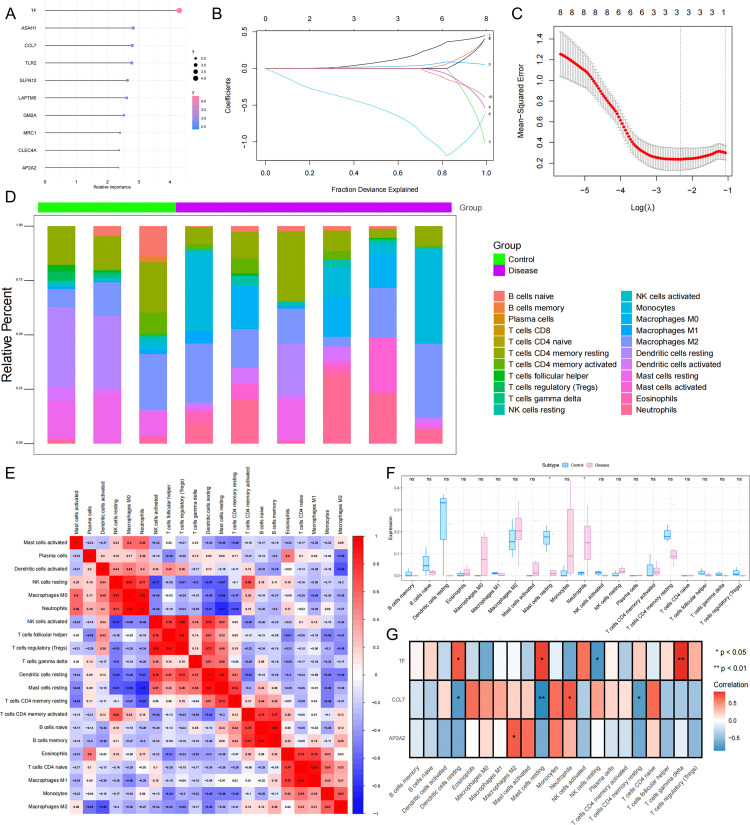
Comparative Analysis of Single-Cell RNA Sequencing Data from Disease and Control Groups. A. Key genes for burn injury identified by random forest screening. B-C. LASSO Regression Analysis. D-E. Immune Cell Proportion and Correlation Heatmaps Among Different Patients. F. Boxplots of Differences in Immune Cell Subpopulations Between Disease and Healthy Control Groups. G. Correlation of TF, CCL7, and AP2A2 Expression in Immune Cells.

To further identify key genes influencing burn injury, we used the FindAllMarker function in single-cell data to calculate differentially expressed genes between cells, extracted macrophage marker genes, and filtered them based on avg_log2FC > 1 and p_val_adj < 0.05. A total of 155 marker genes were identified (Findall.markers.cellType_1.csv). After homologous conversion of human and mouse genes to differentially expressed genes in key cells, we used random forest analysis to identify signature genes in burns. The top 10 genes were identified as burn signature genes (Fig 3A). We then performed Lasso regression on these 10 signature genes, identifying three genes as burn signature genes (Fig 3B, C). These three genes will serve as key genes for our subsequent research: AP2A2, CCL7, and TF.

### Immune infiltration

The microenvironment, primarily composed of immune cells, extracellular matrix, various growth factors, inflammatory factors, and specific physicochemical characteristics, significantly influences disease diagnosis and clinical treatment sensitivity. This study analyzed the relationship between key genes and immune infiltration in a burn dataset to further explore the potential molecular mechanisms by which key genes influence burn progression. This study demonstrated the proportion of immune cells in each patient and the correlation between immune cells in different formats (Fig 3D, E). Furthermore, this study found significant differences in resting mast cells and neutrophils between the two groups (Fig 3F). We further explored the relationship between key genes and immune cells and found that AP2A2 was significantly positively correlated with Macrophage M2. CCL7 was significantly positively correlated with neutrophils and negatively correlated with T cell CD4 memory resting, dendritic cell resting, and mast cell resting. TF was significantly positively correlated with T cell gamma delta, dendritic cell resting, and mast cell resting, and negatively correlated with NK cell resting (Fig 3G).

### Immune regulatory factors

**Fig 4:**
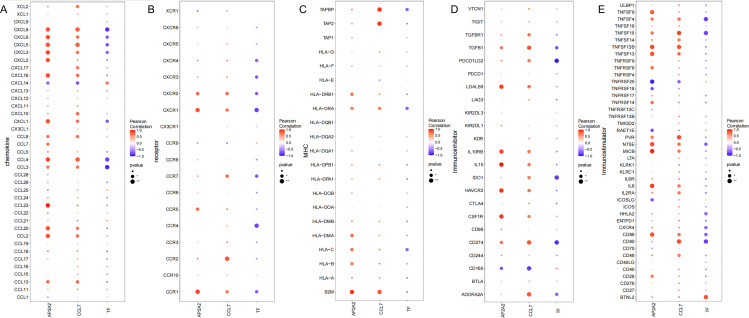
Correlation Between Key Genes and Different Cytokines. A. Correlation of TF, CCL7, and AP2A2 with Chemokines. B. The correlation between TF, CCL7, and AP2A2 with receptors. C. The relationship between TF, CCL7, and AP2A2 with the Major Histocompatibility Complex. D. Correlation between TF, CCL7, and AP2A2 with Immunosuppressive Factors. E. Correlation between TF, CCL7, and AP2A2 with Immunostimulatory Factors.

In addition, we analyzed the correlations between key genes and various immune factors, including immunosuppressive factors, immunostimulatory factors, chemokines, and receptors. These analyses suggest that key genes are closely associated with immune cell infiltration levels and play an important role in the immune microenvironment (Fig 4). Among them, in the chemokine, genes AP2A2 and CCL7 were significantly positively correlated with CXCL8, while genes TF were significantly negatively correlated with CXCL8. In the receptor, genes AP2A2 and CCL7 were significantly positively correlated with CCR1, while genes TF were significantly negatively correlated with CCR1. In the MHC, genes AP2A2 and CCL7 were significantly positively correlated with HLA-DRA, while genes TF were significantly negatively correlated with HLA-DRA. In the immunoinhibitory, genes AP2A2 and CCL7 were significantly positively correlated with CD274, while genes TF were significantly negatively correlated with CD274. In the immunostimulatory, genes AP2A2 and CCL7 were significantly positively correlated with TNFSF15, while genes TF were significantly negatively correlated with TNFSF15.

### GSEA and GSVA analysis

**Fig 5:**
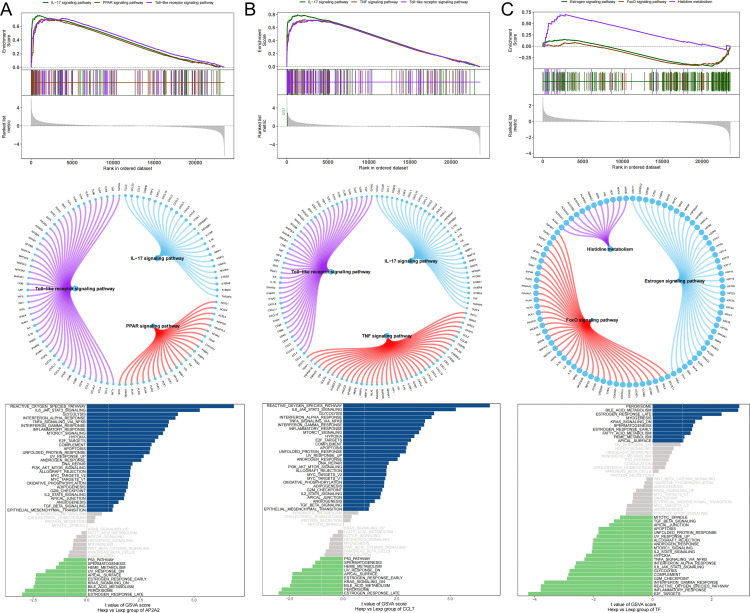
Enrichment Analysis Results of Key Genes. A-C. GSEA and GSVA enrichment analysis results for AP2A2, CCL7, and TF.

Next, we investigated the specific signaling pathways involved in the key genes to explore the potential molecular mechanisms by which these key genes influence disease progression. GSEA results showed that AP2A2 was enriched in signaling pathways such as the IL-17 signaling pathway, the Toll-like receptor signaling pathway, and the PPAR signaling pathway (Fig 5A). CCL7 was enriched in signaling pathways such as the IL-17 signaling pathway, the TNF signaling pathway, and the Toll-like receptor signaling pathway (Fig 5B). TFs were enriched in signaling pathways such as the Estrogen signaling pathway, the Histidine metabolism pathway, and the FoxO signaling pathway (Fig 5C). GSVA analysis showed that AP2A2 was enriched in signaling pathways such as the REACTIVE_OXYGEN_SPECIES_PATHWAY and IL6_JAK_STAT3_SIGNALING pathways (Fig 5A). CCL7 was enriched in signaling pathways such as the REACTIVE_OXYGEN_SPECIES_PATHWAY and IL6_JAK_STAT3_SIGNALING pathways (Fig 5B). TFs were enriched in signaling pathways such as the PEROXISOME and BILE_ACID_METABOLISM pathways (Fig 5C). This suggests that key genes may influence disease progression through these pathways.

### Relationship between key genes and disease-related genes

**Fig 6:**
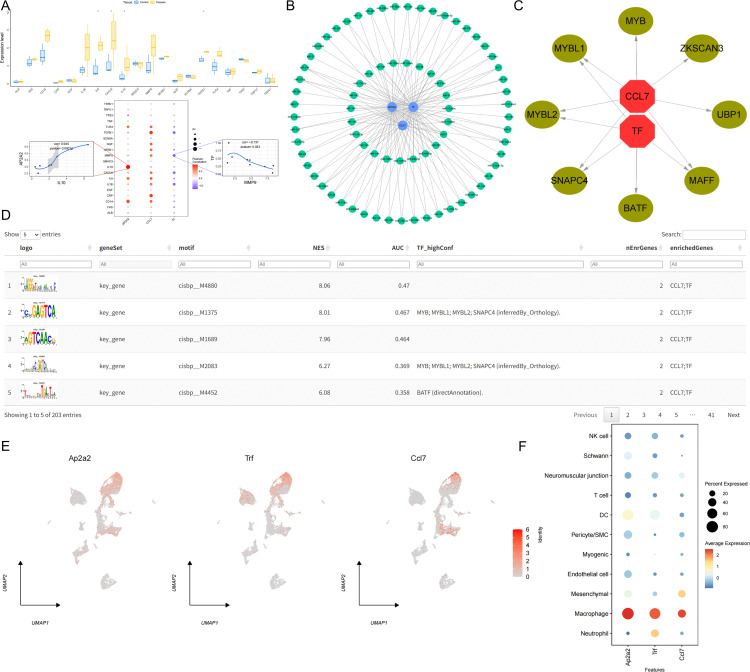
Association Between Gene Expression Analysis and Cell Type Function. A. Gene Expression and Correlation Analysis. B. Network diagram of miRNA interactions with AP2A2, CCL7, and TF. C, D. Visualization of Key Genes and Transcription Factors. E, F. Single-cell analysis of key genes reveals significant expression of key genes in macrophages.

We obtained disease-related genes from the GeneCards database (https://www.genecards.org/). To analyze intergroup expression differences in disease genes, this study analyzed the expression levels of 20 genes with high Relevance* scores and transcriptome expression. We found significant differences in the expression of IL6, CXCL8, IL10, and TGFB1 between the two patient groups. Furthermore, we performed correlation analysis between key genes and disease-related genes. The expression levels of key genes were significantly correlated with those of disease-related genes, with a significant positive correlation between AP2A2 and IL10 (r = 0.945); and a significant negative correlation between TF and MMP9 (r = −0.737) (Fig 6A).

### Key gene miRNA and transcription factor regulatory network

We then used the mircode database to reversely predict key genes, identifying 76 miRNAs and 133 mRNA-miRNA pairs, which were visualized using Cytoscape (Fig 6B). We selected key genes as the gene set for this analysis and found that they are regulated by multiple transcription factors, including common mechanisms. We then performed enrichment analysis of these transcription factors using cumulative recovery curves. Motif-TF annotation and selection analysis of important genes revealed that the motif with the highest normalized enrichment score (NES: 8.06) was cisbp__M4880. We present all enriched motifs and corresponding transcription factors for key genes (Fig 6C, D).

### Single-cell expression profiles of key genes

**Fig 7:**
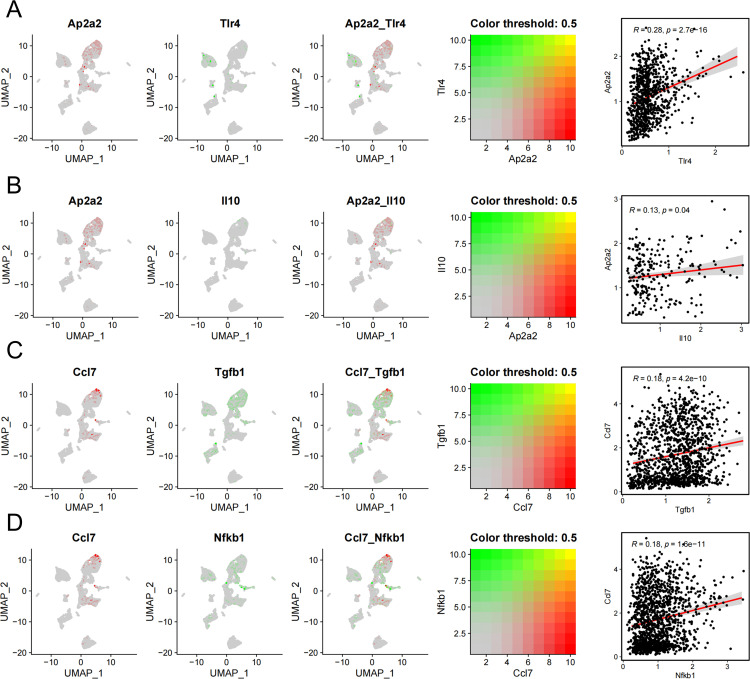
Co-expression Network of Key Genes. A. Co-expression Network of TLR4 and AP2A2. B. Co-expression Network of IL10 and AP2A2. C. Co-expression Network of TGFB1 and CCL7. D. Co-expression Network of NFKB1 and CCL7.

**Fig 8:**
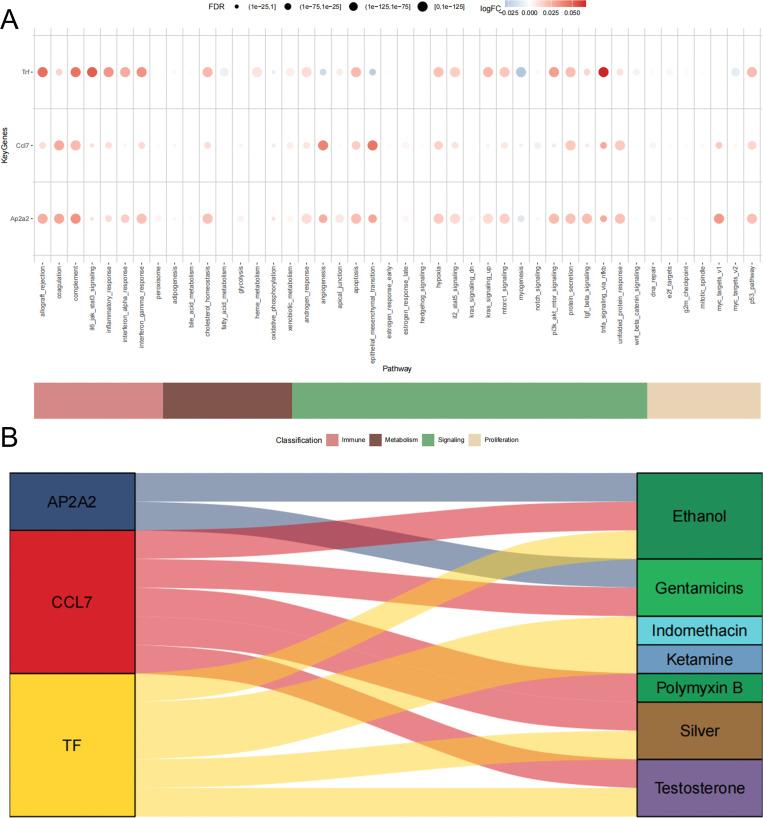
Association Analysis Between Gene Expression and Drugs. A. Enrichment analysis results of key genes (AP2A2, CCL7, TF) across different biological pathways. B. Sanky Diagrams of AP2A2, CCL7, TF with Various Compounds.

This study analyzed the expression levels of key genes in 11 cell types, revealing that Ap2a2, Trf, and Ccl7 were particularly prominent in macrophages (Fig 6E, F). Subsequently, we selected disease genes with high significant correlations from the disease correlation analysis and used correlation analysis to explore the co-expression network between key genes and disease-related genes (Fig 7). We then used the AUCell function to quantitatively analyze the immune and metabolic pathways in the single-cell data, and used bubble plots to visualize the activity differences of key genes in pathways related to immune metabolism. The results showed that Ap2a2, Trf, and Ccl7 had higher activity in pathways such as complement (Fig 8A).

### Drug prediction

In this study, we explored the prediction of targeted drugs for AP2A2, CCL7, and TF based on the CTD public database (https://ctdbase.org/). Our results showed that AP2A2 has two targeted drugs for burns, including ethanoyl and gentamicins; CCL7 has five targeted drugs for burns, including ethanoyl, gentamicins, polymyxin B, silver, and testosterone; and TF has five targeted drugs for burns, including ethanoyl, indomethacin, ketamine, silver, and testosterone (Fig 8B).

## Discussion

Burns represent a profound form of trauma that imposes considerable physiological and psychological stresses on individuals, frequently accompanied by intricate immune responses and a myriad of complications [15–17]. Following a burn injury, the body’s immune and inflammatory mechanisms are markedly amplified. This amplification is closely associated with the expression of a diverse array of cytokines and enzymes [18]. In the aftermath of a burn, the interplay between macrophages and neutrophils plays a pivotal role in the immune response. Macrophages serve as essential immune effector cells. They orchestrate the chemotactic movement of neutrophils by secreting various cytokines and chemokines after the injury [19]. Depending on the microenvironment, macrophages can adopt distinct phenotypic expressions, such as M1 and M2 types. M1 macrophages are linked to pro-inflammatory activities, while M2 macrophages are associated with anti-inflammatory and reparative functions. The variation in macrophage phenotypes can significantly influence the prognosis of patients suffering from burns [20,21]. The regulation of the inflammatory response following a burn is inherently complex. The interaction between macrophages and neutrophils plays a critical role in sustaining immune equilibrium and facilitating wound healing [13,22]. Therefore, immune modulation involving these cellular players after a burn is crucial for ensuring favorable patient outcomes.

**Fig 9:**
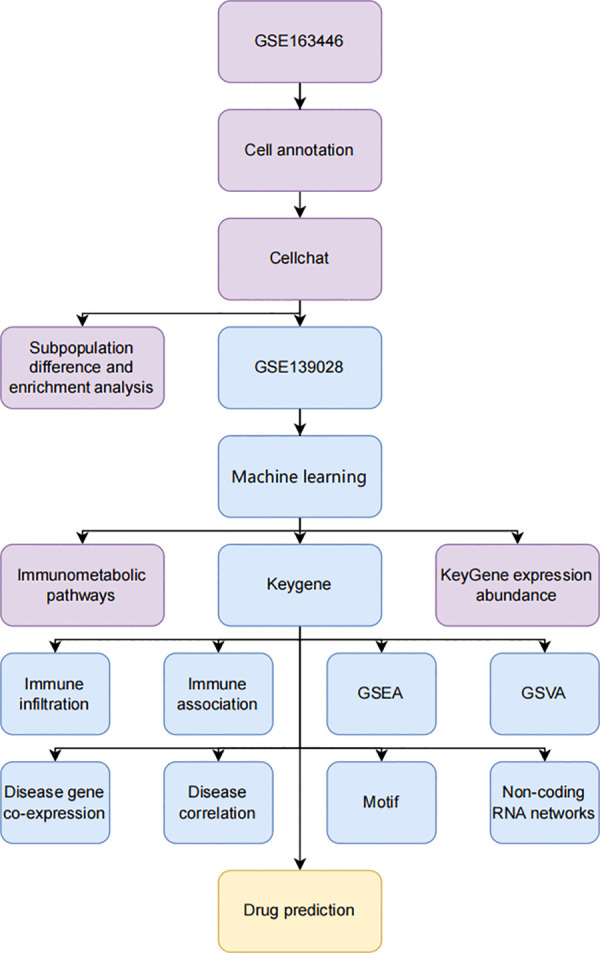
Flowchart.

In recent years, the swift advancement of single-cell sequencing and transcriptomics has yielded unparalleled insights into the underlying mechanisms of burn pathology. This innovative technology has not only unveiled the heterogeneity of immune cell populations but has also facilitated the identification of crucial molecular markers [23–25]. Our differential analysis result sheds light on cell dynamics and immune interactions in the context of burns. Utilizing LASSO regression and the random forest algorithm, we pinpointed 155 macrophage marker genes and highlighted three pivotal genes (AP2A2, CCL7, and TF). Moreover, our investigations revealed significant disparities in immune cell proportions, particularly concerning mast cells and neutrophils. Research conducted by Song J, and colleagues underscores that the interplay between various macrophage subpopulations and other cell types is instrumental in modulating inflammation and fostering wound healing [26]. In recent years, numerous studies have explored strategies to improve wound healing through biomaterials for burn injuries. For instance, Chen reported a multi-layer microneedle patch that promoted angiogenesis and regulated the local immune microenvironment in a thermal burn model, thereby enhancing healing quality while reducing scar formation [27]. Similarly, CuCS/Cur composite electrospun dressings facilitated regeneration of skin appendages—including nerves, blood vessels, and hair follicles—in deep burn injuries, underscoring the potential of biomaterials for functional skin reconstruction [28]. Conditional effects analysis revealed that AP2A2 and CCL7 exhibited positive correlations with M2 macrophages and neutrophils, respectively; while TF demonstrated a negative correlation with NK cells. Altering the macrophage phenotype can profoundly improve the healing environment in burn wounds by modulating the secretion of inflammatory factors [29,30]. Following burn injuries, the activation of signaling pathways such as NF-κB and MAPK can also impact the polarization of macrophages. This, in turn, influences the prognosis for burn patients [31]. Consequently, targeting the intercellular signaling pathways presents an opportunity to adjust macrophage polarization, ultimately enhancing patient outcomes [32]. The high correlation coefficients, notably the strong positive correlation between AP2A2 and IL10 (r = 0.945), indicate a robust connection that could significantly influence the inflammatory response and healing processes in burns. Additionally, the negative correlation between TF and MMP9 (r = −0.737) suggests a potential regulatory mechanism, where TF may inhibit MMP9 expression, a gene recognized for its role in extracellular matrix remodeling and wound healing (Fig 9).

In our research, we face several constraints that warrant attention. Firstly, the analysis relied on publicly accessible data, and we lack independent datasets and experimental validation to ascertain the robustness of our findings. Secondly, while our investigation highlighted crucial genes (AP2A2, CCL7, and TF), clinical validation is necessary to establish their clinical utility and accuracy. Although the present study identified relevant signaling pathways using approaches such as GO and KEGG enrichment analysis, it lacks ROC curve analysis to validate the prognostic value of these genes, as well as mechanistic experiments to confirm their role as therapeutic targets. Relevant genes and pathways identified in the present study via bioinformatics approaches have not been validated by relevant experiments, and their functional associations with burn injury require further experimental verification; nevertheless, the current phase of this research provides potential candidate genes and signaling pathways for future investigations. Importantly, Chen and Zhang have demonstrated that the possibility of improving the quality of wound healing through advanced biomaterials. These studies complement the immune regulation and key gene action mechanism proposed in this research, further indicating that future burn treatment can be advanced in a coordinated manner through both molecular regulation and material intervention [27,28]. Therefore, the integration of clinical data, along with the conduct of well-designed prospective clinical studies, could yield invaluable insights into the real-world applicability and efficacy of these genes. This study concentrated on bioinformatics analysis, laying the foundation for future experimental validation of key genes and pathways related to burns, functional characterization, and mechanistic investigations. Furthermore, we identified potential targeted therapies for these key genes through the Comparative Toxicogenomics Database (CTD), providing promising options for future clinical applications. Subsequent experimental research should aim to clarify the biological significance of these genes, investigate their interactions within cellular processes, and assess potential therapeutic targets. Overall, as technology continues to evolve, single-cell sequencing and transcriptomics are revolutionizing our comprehension of burn pathology. Researchers progressively uncover essential biomarkers through multi-omics integration studies, revealing the intricate communication networks among cells [33,34]. Looking forward, with the ongoing refinement of data analysis techniques and a deeper understanding of biological mechanisms, research in burn treatment is poised to achieve significant breakthroughs, offering more effective therapeutic options for patients.

## Conclusion

In this study, we delved into the single-cell landscape of burns and accurately pinpointed three key genes: AP2A2, CCL7, and TF. We comprehensively unveiled the intricate network of intercellular communication and precisely mapped the panorama of immune cell infiltration, successfully identifying macrophages as the key cell subpopulation. Through rigorous signal pathway enrichment analysis, we found these genes to be closely associated with key signaling pathways such as IL-17 and Toll-like receptors, offering new insights into the molecular mechanisms of burn progression. Moreover, in-depth drug prediction analysis led us to identify potential targeted drugs for these three genes, paving the way for new therapeutic targets and research directions for burn treatment. This achievement not only deepens the understanding of the burn immune microenvironment but also provides invaluable references for clinical treatment.

## Supporting information

S1 Fig Quality Control Analysis of Single-Cell RNA Sequencing Data. A. Violin plots evaluating sequencing data quality across different samples. B. Scatter plots further analyzed the relationships among these indicators.
(TIF)

S2 Fig Variability Analysis and Batch Effect Correction of Single-Cell RNA Sequencing Data. A. Analyzed 2,000 highly mutated genes and presented the top 10 genes. B. Principal Component Analysis (PCA) Scatterplot. C. Two-dimensional scatter plot of data after dimensionality reduction.
(TIF)
